# Computer self-efficacy and technostress as independent predictors of job burnout among community healthcare professionals: a cross-sectional study

**DOI:** 10.3389/fpubh.2026.1835397

**Published:** 2026-05-21

**Authors:** Yi Wu, Qing Ma, Hong Liu, Ronghua Fang

**Affiliations:** General Practice Ward, International Medical Center Ward, General Practice Medical Center, West China Hospital/West China School of Nursing, Sichuan University, Chengdu, China

**Keywords:** community healthcare workers, computer self-efficacy, job burnout, job demands-resources model, occupational health, technostress

## Abstract

**Background:**

The digital transformation of healthcare has introduced new occupational stressors—including technostress—that may contribute to job burnout among community healthcare professionals. This study aimed to investigate how technostress and computer self-efficacy influence job burnout, specifically determining if they act through independent pathways or mediation.

**Methods:**

A cross-sectional survey was conducted among 329 community healthcare professionals between November and December 2025. Data were collected using the Computer Self-Efficacy Scale, the Technostress Scale, and the Maslach Burnout Inventory-General Survey. Structural equation modeling and bootstrapping with 5,000 resamples were employed to analyze path relationships and examine mediation effects.

**Results:**

Computer Self-Efficacy exerted significant negative direct effects on exhaustion and cynicism, and a positive effect on professional efficacy (all *p* < 0.001). Conversely, technostress showed the opposite pattern, increasing exhaustion and cynicism while reducing professional efficacy (all *p* < 0.001). Computer Self-Efficacy did not significantly affect technostress (*p* = 0.71), ruling out a mediation effect.

**Conclusion:**

Computer Self-Efficacy and technostress affect job burnout through distinct, independent pathways. High Computer Self-Efficacy protects workers psychologically but cannot neutralize the stress caused by system design and structural factors. To reduce burnout in digital healthcare settings, organizational-level interventions must go beyond individual training and actively improve the usability of health information systems.

## 1 Introduction

While digital transformation is vital for improving primary healthcare quality (1), introducing systems like Electronic Health Records in resource-constrained institutions expands work boundaries and increases cognitive load (2). Prior research has primarily focused on organizational efficiency gains (3). However, integrating technology into healthcare can intensify existing challenges and trigger technostress among professionals (4). Unresolved stress may lead to job burnout and impair physical and mental wellbeing, thereby obstructing the development of a sustainable work environment (2, 5). Given the significant individual differences in digital adaptability (6), personal resources likely influence these outcomes alongside technostress.

According to the Job Demands-Resources (JD-R) model (7), burnout results from the interaction between demands and resources, where personal resources exert a direct beneficial effect and may also buffer the impact of job demands. Computer Self-Efficacy (CSE), as a key resource, has been shown to reduce stress and burnout (8). Thus, CSE likely provides similar protection against technostress and burnout among community healthcare professionals.

Drawing on the JD-R model (7), technostress is defined as a typical hindrance job demand (9, 10). Community healthcare professionals face frequent system updates, interface complexity, and multi-platform data entry, resulting in a heavy information processing burden (3, 11). The health impairment hypothesis of the JD-R model posits that continuous exposure to such rigid demands depletes psychological and physiological energy without adequate recovery, leading to resource exhaustion (7). This continuous loss of energy is causally linked to job burnout (12). Therefore, as a specific environmental stressor, technostress constitutes a critical risk factor for job burnout among community healthcare professionals.

The JD-R model emphasizes the independent protective role of personal resources in occupational health (7, 13). In digital environments, CSE is regarded as a vital personal resource, defined as “a judgment of one's capability to use a computer” (14). According to Bandura's Social Cognitive Theory (15), self-efficacy reflects an individual's belief in their ability to execute tasks required to manage prospective situations. Building on this, Compeau et al. (16) conceptualized CSE as a domain-specific form of self-efficacy, capturing individuals' confidence in performing computer-related tasks in specific technological contexts. Conceptually, CSE aligns with digital resilience, representing a sense of mastery and confidence in coping with digital challenges (17). According to Conservation of Resources (COR) theory (18), this sense of control enriches personal resources, enabling individuals to resist resource loss and potentially blocking burnout.

Currently, there is a lack of consensus in the academic community regarding how personal resources mitigate job burnout caused by technostress specifically within the community healthcare context. Based on the buffering hypothesis of the JD-R model, previous studies have often expected personal resources (such as self-efficacy) to buffer or reduce the negative impact of technostress, thereby blocking its effect on job burnout (i.e., the stress reduction–burnout prevention pathway) (13, 19). However, evidence varies across contexts. First, at the level of stress perception, Li et al. (20) demonstrated that high levels of digital competence significantly lower how individuals perceive technostress in the maritime industry. Second, at the level of burnout buffering, previous evidence (8) suggests that CSE can serve as an effective buffer against general work stress, significantly reducing job burnout. Evidence from healthcare settings is limited. Although technology competence has been found to weaken the association between technostress and burnout (21), its applicability to community healthcare remains unclear.

According to the JD-R model, high levels of personal resources can also exert a direct beneficial effect by initiating a motivational process (7, 13). Nevertheless, in community healthcare settings characterized by rigid administrative constraints and high workloads (2, 11, 22), simply improving individual confidence may not alter objective system complexity. It remains unclear whether CSE primarily affects burnout indirectly by reducing perceived technostress or acts as an independent resource exerting a direct compensatory effect. Clarifying these pathways is practically important to determine whether future occupational health interventions should focus on individual digital skills training or necessitate broader ergonomic system redesigns.

Drawing on the JD-R model and COR theory, this study frames technostress as a hindrance demand and CSE as a critical personal resource. As an external stressor, technostress may trigger a resource loss process, depleting psychological energy and ultimately leading to job burnout (5, 9, 12). Conversely, high CSE enables individuals to protect and acquire resources (8, 16, 17), mitigating the perceived threat of technological demands and preventing excessive resource depletion. Integrating these perspectives, this study examines both the direct (resource compensation) and indirect (mediation) pathways through which CSE and technostress influence burnout.

Guided by the proposed model, the aim of this study was to investigate the relationships among CSE, technostress, and job burnout in community healthcare professionals. Specifically, the study sought to clarify the pathways through which CSE and technostress influence burnout. On the basis of these aims, the following hypotheses are formulated: H1: CSE negatively predicts technostress; H2: technostress positively predicts job burnout; H3: CSE negatively predicts job burnout; H4: technostress mediates the relationship between CSE and job burnout.

## 2 Materials and methods

### 2.1 Study design and participants

A cross-sectional survey was conducted to examine the associations among CSE, technostress, and job burnout in Chinese community healthcare professionals. Guided by the JD-R model, the study was reported in accordance with the STROBE guidelines. Participants were recruited from community health service centers in [Province Name Omitted for Blind Review], China, between November and December 2025, using convenience and snowball sampling methods, and routinely using digital systems such as electronic health records and public health platforms in their daily work.

### 2.2 Data collection

Data were collected via an online survey platform (Wenjuanxing). The survey link and QR code were distributed to liaisons at each center via WeChat work groups. To ensure data quality, only healthcare professionals within the participating centers were eligible for inclusion. Several control measures were implemented: (1) all items were set as mandatory to prevent missing data; (2) each IP address or WeChat account was restricted to a single submission; and (3) questionnaires completed in under 60 s or exhibiting uniform response patterns (e.g., straight-lining) were excluded during data cleaning. No personally identifiable information was collected or retained; all data were de-identified prior to analysis.

Based on the recommendation by Hair et al. (23), the sample size for Structural Equation Modeling (SEM) requires a sample size 5 to 10 times the number of observed variables, the minimum target for the 33-item measurement model was established at 165 to 330 participants. A total of 338 questionnaires were returned, yielding 329 valid responses (effective response rate: 97.3%). To verify sensitivity, a power analysis was conducted using G^*^Power 3.1; with α = 0.05, effect size (*f*^2^) = 0.15 (medium), and 7 predictors, the statistical power of the obtained sample (*N* = 329) exceeded 0.99.

### 2.3 Measures

#### 2.3.1 Demographic questionnaire

A self-designed questionnaire was used to collect demographic information, including gender, age, professional role, work experience, professional title, educational level, management position, monthly income, practice setting (urban/rural), daily system use, weekly work hours, permanent position, marital status, and number of children.

#### 2.3.2 Computer self-efficacy scale

CSE was measured using the scale originally developed by Compeau and Higgins (14). The Chinese version translated by Shi Meng (24) was utilized, with minor linguistic adaptations incorporated to suit the healthcare context. The 10-item instrument assesses individuals' confidence in their ability to execute specific computer tasks (e.g., “I could complete the job using the system if there was no one around to tell me what to do as I go”). Items are rated on a 5-point Likert scale ranging from 1 (strongly disagree) to 5 (strongly agree), where higher total scores reflect greater CSE. The scale has demonstrated high internal consistency in prior research, with a Cronbach's α of 0.943 (24).

#### 2.3.3 Technostress scale

Technostress was measured using the scale originally developed by Tarafdar et al. (25). A validated Chinese version (26) was utilized, with wording adapted for the community healthcare context. To maximize relevance to clinical practice, the 8-item “Techno-overload” and “Techno-complexity” dimensions were selected (e.g., “I am forced by this technology to work much faster”). The two dimensions were combined into a single composite score, with higher scores indicating greater technostress. Items were rated on a 5-point Likert scale ranging from 1 (strongly disagree) to 5 (strongly agree), where higher scores reflect greater perceived technostress. Previous research reported a Cronbach's α of 0.85 for this instrument (26).

#### 2.3.4 Maslach burnout inventory-general survey (MBI-GS)

Job burnout was assessed using the Chinese version of the MBI-GS (27, 28). This instrument evaluates three dimensions: exhaustion (five items), Cynicism (four items, reflecting depersonalization in healthcare), and Professional Efficacy (six items). Responses are scored on a 7-point Likert scale ranging from 0 (never) to 6 (every day). Elevated scores in Exhaustion and Cynicism, coupled with reduced scores in Professional Efficacy, indicate higher levels of burnout. In this study, the three dimensions were analyzed separately to capture different aspects of burnout. The scale demonstrated good internal consistency in previous studies, with a Cronbach's α of 0.868 (28).

#### 2.3.5 Instrument adaptation and pre-test

Established Chinese versions of the scales were adapted to the community healthcare context with minor wording adjustments to reflect clinical and digital workflows. A pre-test with five healthcare professionals was conducted to assess clarity, readability, and contextual relevance, and minor revisions were made accordingly. This pre-test was limited to face validity and linguistic appropriateness; psychometric properties were evaluated using the full sample (*N* = 329).

### 2.4 Data analysis

Data analysis was performed using IBM SPSS Statistics 26.0 and IBM SPSS AMOS 26.0. Continuous variables conforming to a normal distribution (e.g., CSE, technostress, and job burnout scores) were described using means and standard deviations (M ± SD), while categorical variables were described using frequencies and percentages (*n*, %). Pearson correlation analysis was used to examine the relationships among major variables.

Potential common method bias was evaluated using Harman's single-factor test, supplemented by the Heterotrait-Monotrait ratio (HTMT) ratio to assess construct redundancy. Subsequently, Confirmatory Factor Analysis (CFA) was performed using AMOS 26.0 to verify the construct validity of the scales. The criteria for model fit were set as: χ^2^/*df* < 5, CFI > 0.90, TLI > 0.90, RMSEA < 0.10 (29). During model optimization, based on modification indices, partial residual correlations within the same latent variable were permitted to improve model fit.

Finally, SEM was constructed to test the research hypotheses. To control for potential confounding effects of demographic characteristics, age, work experience, monthly income, marital status, and number of children were included as control variables in the model to account for their potential influence on technostress and job burnout dimensions. The Bootstrap method (with 5,000 resampling iterations) was employed to test the significance of mediation effects by calculating 95% confidence intervals (CI). If the 95% CI did not include 0, the mediation effect was considered significant; otherwise, it was not. All statistical tests were two-tailed, with *p* < 0.05 indicating statistical significance.

### 2.5 Ethical considerations

Ethical approval was obtained from the Biomedical Ethics Committee of West China Hospital, Sichuan University (Approval No. 2025-2624). Participants reviewed an online consent statement covering the study aims and anonymity prior to participation. Completing the questionnaire served as implied consent, and all data remained anonymized and confidential for research use only.

## 3 Results

### 3.1 Common method bias test

Common Method Bias (CMB) was evaluated using Harman's single-factor test. The primary extracted factor accounted for 33.16% of the variance, well below the 50% threshold (30). Additionally, discriminant validity was assessed to examine whether method variance could artificially inflate construct relationships. All HTMT values (0.05–0.75) were below the conservative 0.85 criterion (31), confirming construct distinctiveness and further mitigating concerns of severe CMB.

### 3.2 Participant characteristics

A total of 329 valid responses were included in the final analysis. The sample consisted of 294 females (89.4%) and 35 males (10.6%). The participants ranged in age from 21 to 68 years, with a mean age of 35.30 ± 7.25 years. Regarding professional roles, community nurses constituted the majority (63.2%), followed by community physicians (13.1%) and other professionals (23.7%). Detailed demographic characteristics are presented in Table 1.

**Table 1:** Demographic characteristics of the participants (*N* = 329).

Characteristics	*n* (%)/M ±SD
Gender	
Male	35 (10.6)
Female	294 (89.4)
Age (years)	35.30 ± 7.25
Marital status	
Unmarried	69 (21.0)
Married/cohabiting	251(76.3)
Divorced/widowed	9 (2.7)
Children	
0	96 (29.2)
1	142 (43.2)
2	89 (27.1)
≥3	2 (0.6)
Practice setting	
Urban	247 (75.1)
Rural	82 (24.9)
Education level	
Technical school or below	2 (0.6)
Associate degree	59 (17.9)
Bachelor's degree	264 (80.2)
Master's degree or above	4 (1.2)
Professional role	
Doctor	43 (13.1)
Nurse	208 (63.2)
Other professionals	78 (23.7)
Management position	
Yes	61 (18.5)
No	268 (81.5)
Permanent position	
Yes	66 (20.1)
No	263 (79.9)
Professional title	
Junior	139 (42.2)
Intermediate	163 (49.5)
Vice-senior	15 (4.6)
Senior	2 (0.6)
Other	10 (3.0)
Monthly income (yuan)	
< 3,000	20 (6.1)
3,000–4,999	132 (40.1)
5,000–6,999	136 (41.3)
7,000–9,999	34 (10.3)
≥10,000	7 (2.1)
Work experience (years)	13.53 ± 7.61
Weekly work hours	
≥40 h	67 (20.4)
51–60 h	57 (17.3)
>60 h	34 (10.3)
Daily system use (h)	6.51 ± 2.83

### 3.3 Descriptive statistics and correlation analysis

Prior to the main analysis, independent samples *t*-tests were conducted to examine potential differences in key variables between the core professional groups (community nurses, *n* = 208 and other healthcare professionals, *n* = 121) to justify data pooling. The results indicated no statistically significant differences between the two groups regarding CSE (*t* = −0.03, *p* = 0.97), Technostress (*t* = 0.07, *p* = 0.95), Exhaustion (*t* = −0.11, *p* = 0.92), Cynicism (*t* = 0.52, *p* = 0.60), and Professional Efficacy (*t* = 0.11, *p* = 0.91). Given the high homogeneity of the data, the samples were pooled (*N* = 329) for subsequent statistical analyses.

Table 2 reports the descriptive statistics and bivariate correlations for all study variables. As anticipated, CSE exhibited negative associations with the two burnout dimensions—Exhaustion (*r* = −0.19, *p* < 0.01) and Cynicism (*r* = −0.30, *p* < 0.01)—while showing a positive relationship with Professional Efficacy (*r* = 0.35, *p* < 0.01). Conversely, Technostress correlated positively with both Exhaustion (*r* = 0.27, *p* < 0.01) and Cynicism (*r* = 0.26, *p* < 0.01), but negatively with Professional Efficacy (*r* = −0.26, *p* < 0.01). Notably, no significant correlation was found between CSE and technostress (*r* = 0.02, *p* > 0.05), which preliminarily supports the rationale for treating them as distinct independent constructs within the model.

**Table 2:** Descriptive statistics and correlations among study variables (*N* = 329).

Variables	Mean	SD	1	2	3	4	5
CSE	3.59	0.86	1				
TS	2.98	0.86	0.02	1			
EX	3.43	1.24	−0.19^**^	0.27^**^	1		
CY	2.81	1.25	−0.30^**^	0.26^**^	0.71^**^	1	
PE	4.55	1.38	0.35^**^	−0.26^**^	−0.12^*^	−0.25^**^	1

CSE, computer self-efficacy; TS, technostress; EX, exhaustion; CY, cynicism; PE, professional efficacy.

All correlations are significant at *p* < 0.01 level (2-tailed), except where noted. CSE and TS used a 5-point Likert scale (1-5); Burnout dimensions (EX, CY, PE) used a 7-point frequency scale (0–6).

Demographic variables were controlled in the analysis but omitted here for brevity.

^*^p < 0.05; ^**^p < 0.01.

### 3.4 Measurement model

#### 3.4.1 Reliability test

Cronbach's α was used to test the internal consistency of the scales. The results showed that the Cronbach's α for CSE and Technostress were 0.973 and 0.972, respectively. For the MBI-GS, the Cronbach's α for the three dimensions were 0.955 (Exhaustion), 0.943 (Cynicism), and 0.930 (Professional Efficacy). All coefficients indicated strong internal consistency. While exceptionally high values (α > 0.95) can conceptually imply item redundancy (23), this methodological implication was proactively addressed during model refinement by removing items with insufficient factor loadings and specifying correlated errors for items with explicit semantic overlap.

#### 3.4.2 Validity test

CFA was conducted using AMOS 26.0. During model refinement, two items from the Professional Efficacy dimension (C10 and C11) were removed due to low standardized factor loadings (< 0.50). Additionally, five pairs of error covariances were specified within the same latent variables based on modification indices and similarities in item wording or content (e.g., items describing task performance without assistance). These modifications were limited to maintain model parsimony and avoid overfitting. The refined five-factor measurement model demonstrated an acceptable fit: χ^2^/*df* = 3.352, CFI = 0.913, TLI = 0.901, and RMSEA = 0.085. The RMSEA value indicates a slightly elevated but acceptable level of approximation error (29).

Regarding convergent validity (see Table 3), the standardized factor loadings for all retained items ranged from 0.69 to 0.98 (all *p* < 0.001). Both Composite Reliability (CR) and Average Variance Extracted (AVE) values exceeded established benchmarks (23), with CR estimates spanning 0.93 to 0.98 and AVE ranging from 0.77 to 0.83. Collectively, these parameters substantiate the structural integrity of the measurement tool.

**Table 3:** Reliability and validity assessment of the measurement model.

Construct	Item	Std. factor loading	Cronbach's α	CR	AVE
CSE TS	A1	0.84	0.97 0.97	0.98 0.97	0.82 0.80
	A2	0.87			
	A3	0.90			
	A4	0.89			
	A5	0.88			
	A6	0.88			
	A7	0.80			
	A8	0.90			
	A9	0.93			
	A10	0.93			
	B1	0.76			
	B2	0.81			
	B3	0.81			
	B4	0.84			
	B5	0.90			
	B6	0.98			
	B7	0.97			
	B8	0.96			
EX	C1	0.91	0.96	0.96	0.82
	C2	0.87			
	C3	0.92			
	C4	0.96			
	C5	0.84			
CY	C6	0.91	0.94	0.95	0.83
	C7	0.96			
	C8	0.90			
	C9	0.82			
PE	C12	0.69	0.93	0.93	0.77
	C13	0.93			
	C14	0.96			
	C15	0.92			

CSE, computer self-efficacy; TS, technostress; EX, exhaustion; CY, cynicism; PE, professional efficacy; CR, composite reliability; AVE, average variance extracted; Std. factor loading, standardized factor loading.

All factor loadings are significant at *p* < 0.001.

Discriminant validity was assessed using the HTMT (31). As shown in Table 4, all HTMT values (0.05–0.75) were well below the conservative 0.85 threshold, confirming that the constructs are conceptually distinct.

**Table 4:** Heterotrait-Monotrait ratio (HTMT) for discriminant validity.

Constructs	CSE	TS	EX	CY	PE
CSE	—				
TS	0.05	—			
EX	0.19	0.28	—		
CY	0.32	0.27	0.75	—	
PE	0.41	0.27	0.19	0.33	—

CSE, computer self-efficacy; TS, technostress; EX, exhaustion; CY, cynicism; PE, professional efficacy.

HTMT values < 0.85 indicate established discriminant validity (31).

### 3.5 Structural model results

The SEM was examined to test the proposed research hypotheses. The path coefficients and significance levels are presented in Table 5.

**Table 5:** Path coefficients of the structural model.

Hypothesis	Path	Std. (β)	*P*-value	Result
H1	CSE → TS	−0.02	0.710	Not supported
H2a	TS → EX	0.25	^***^	Supported
H2b	TS → CY	0.24	^***^	Supported
H2c	TS → PE	−0.23	^***^	Supported
H3a	CSE → EX	−0.20	^***^	Supported
H3b	CSE → CY	−0.29	^***^	Supported
H3c	CSE → PE	0.45	^***^	Supported
Control variable	Income → TS	−0.12	^*^	Significant

CSE, computer self-efficacy; TS, technostress; EX, exhaustion; CY, cynicism; PE, professional efficacy.

Effects of other control variables (age, work experience, marital status, and children) were tested but found to be non-significant (*p* > 0.05) and are omitted for brevity.

^***^p < 0.001, ^*^p < 0.05.

#### 3.5.1 Direct effects

**Figure 1:**
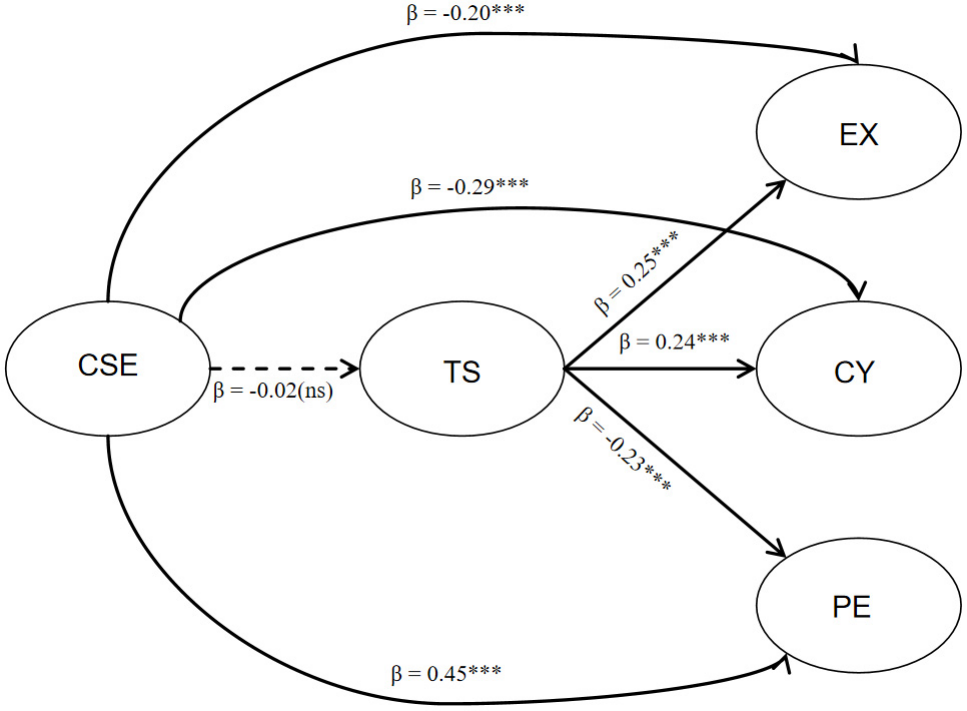
Path analysis results of the structural equation model. CSE, computer self-efficacy; TS, technostress; EX, exhaustion; CY, cynicism; PE, professional efficacy. Ellipses represent latent variables. Values on the arrows are standardized path coefficients (β). Solid lines indicate significant paths, while the dashed line indicates a non-significant path. ***p < 0.001, ns, not significant.

The results of the path analysis (see Figure 1 and Table 5) indicated that: hypothesis 1: CSE did not significantly predict technostress (β = −0.02, *p* = 0.71). Consequently, Hypothesis 1 was not supported. Hypothesis 2: supporting Hypothesis 2, technostress exhibited significant positive predictive effects on Exhaustion (β = 0.25, *p* < 0.001) and Cynicism (β = 0.24, *p* < 0.001), alongside a negative predictive effect on Professional Efficacy (β = −0.23, *p* < 0.001). Hypothesis 3: CSE exerted significant negative direct effects on Exhaustion (β = −0.20, *p* < 0.001) and Cynicism (β = −0.29, *p* < 0.001), while robustly enhancing Professional Efficacy (β = 0.45, *p* < 0.001). These findings fully support Hypothesis 3.

Control Variables: among the included covariates, higher monthly income was uniquely and significantly associated with lower perceived technostress (β = −0.12, *p* < 0.05).

#### 3.5.2 Indirect effects

A 5,000-resample bootstrapping procedure was utilized to evaluate the mediating role of technostress (Table 6). The 95% confidence intervals (CIs) for the indirect effects of CSE via technostress on Exhaustion ([−0.060, 0.068]), Cynicism ([−0.059, 0.065]), and Professional Efficacy ([−0.083, 0.061]), all encompassed zero, indicating non-significant indirect pathways. Consequently, Hypothesis 4 was not supported. However, the total effect analysis indicated that the total effects of CSE on all dimensions of job burnout were statistically significant. This suggests that in the community healthcare context, CSE protects against job burnout primarily through direct pathways rather than by mitigating technostress.

**Table 6:** Analysis of mediation effects.

Pathways	Std. Effect	Boot SE	95% LLCI	95% ULCI
Exhaustion				
Total effect (CSE → EX)	−0.268	0.078	−0.421	−0.114
Indirect effect (CSE → TS → EX)	0.008	0.032	−0.060	0.068
Cynicism				
Total effect (CSE → CY)	−0.436	0.076	−0.586	−0.286
Indirect effect (CSE → TS → CY)	0.008	0.031	−0.059	0.065
Professional efficacy				
Total effect (CSE → PE)	0.556	0.083	0.393	0.719
Indirect effect (CSE → TS → PE)	−0.009	0.036	−0.083	0.061

CSE, computer self-efficacy; TS, technostress; EX, exhaustion; CY, cynicism; PE, professional efficacy; CI, confidence interval; LL/UL, lower/upper limit.

Point estimate: unstandardized regression coefficients.

Boot SE: standard errors based on 5,000 bootstrap samples.

Unstandardized regression coefficients are reported.

Indirect effects are statistically significant if the 95% CI excludes zero.

## 4 Discussion

Addressing job burnout remains a critical priority for healthcare quality and safety (32, 33), particularly as digital transformation exposes healthcare professionals to unprecedented pressures of technological adaptation (4). Framed within the JD-R model (7), the current findings challenge initial theoretical expectations: the anticipated mediation pathway (Hypothesis 4) was unsupported. Rather than CSE buffering the perception of technostress, an independent, dual-pathway structure emerged. CSE operates as a vital personal resource directly mitigating burnout; simultaneously, technostress functions as a persistent hindrance demand (10) that independently depletes individual energy.

### 4.1 The independent protective role of CSE

CSE significantly diminishes Exhaustion and Cynicism while robustly enhancing Professional Efficacy. Unlike previous studies which emphasized that self-efficacy indirectly affects work outcomes via coping styles or behavioral pathways (34, 35), this study found no mediating role of CSE via technostress; instead, it demonstrated an independent direct effect. Within the JD-R framework, CSE functions as a critical personal resource. This suggests that in environments where the technological context is relatively fixed and stress is persistent, an individual's sense of digital mastery directly fuels the motivational process, fortifying their psychological resource reservoir without necessitating a reduction in objective stressors. This direct enhancement aligns with the motivational processes of the JD-R model (7). In the digital era, confidence in technological mastery is viewed as a vital intrinsic resource for maintaining professional accomplishment (36). Even amidst persistent environmental pressures—such as cumbersome EMRs or task overload—elevated CSE serves as a psychological anchor, continuously stimulating professional efficacy.

The direct inhibition of Exhaustion and Cynicism by CSE substantiates the resource caravan perspective of COR theory (18), which posits that individuals possessing abundant positive resources are less susceptible to stress depletion. As Liu et al. (35) noted, self-efficacy transcends mere operational ability to function as a vital psychological buffer—a role that CSE assumes in the current digital context through two distinct cognitive-behavioral mechanisms.

First, regarding problem-focused coping, individuals invest existing resources to block further stress depletion (18). Healthcare professionals exhibiting high CSE actively channel psychological resources into confronting, rather than avoiding, technostress. Consistent with Atiq et al. (37), who found that high self-efficacy promotes superior active coping strategies, elevated CSE drives individuals to solve technical problems head-on. This proactive engagement may help limit resource depletion and is negatively associated with Exhaustion.

Second, high CSE endows individuals with greater cognitive flexibility, facilitating effective cognitive reappraisal. Notably, a strong sense of competence is recognized as a key antecedent for activating such strategies to improve emotional states (38). In community healthcare settings, this flexibility prompts professionals to reframe technical difficulties—such as system failures—as manageable challenges rather than uncontrollable threats. Maintaining this “sense of control” attenuates helplessness and prevents the accumulation of negative emotions. By effectively containing resource depletion, CSE prevents the onset of an emotional exhaustion loss spiral, thereby significantly mitigating job burnout even amidst persistent objective technostress (18, 33).

### 4.2 Technostress as a structural hindrance demand

Conversely, technostress functioned exclusively as a persistent hindrance demand (10), that drives the health impairment process, positively predicting Exhaustion and Cynicism. The lack of a significant association between CSE and technostress highlights a critical structural dilemma in China's community healthcare digital transformation (11). Driven by the need for data standardization, health information systems are typically implemented via a “top-down” approach (39). While beneficial for macro-level integration, this architecture imposes rigid constraints on frontline end-users, who rarely participate in interface design (40). Consequently, when systems prioritize administrative data structuring over clinical usability, the resulting techno-overload stems from architectural flaws rather than competence-based deficiencies (11, 40, 41). This systemic rigidity may exceed the regulatory capacity of personal resources. From a JD-R perspective, this helps explain why CSE and technostress operate as independent pathways: certain structural job demands, particularly those embedded in system design, are less amenable to individual mitigation. This finding suggests that the buffering role of personal resources against technostress may be constrained by specific organizational contexts (42).

This diverges from general occupational findings, where CSE serves as a direct antecedent capable of reducing technostress (43). This inconsistency suggests that the relationship between CSE and technostress may be context-specific. In community healthcare settings, where system design is relatively rigid and user autonomy is limited, technostress appears to be more closely related to structural constraints than to individual capability. As Konttila et al. (42) observed, inadequate equipment and poorly designed support systems constitute organizational barriers that individual adaptability cannot overcome. Counterintuitively, elevated CSE might even amplify frustration: highly digitally competent professionals more acutely recognize the disconnect between flawed system logic and actual clinical workflows (6). Thus, technostress in this environment represents a structural hindrance stressor (6, 9)—an objective obstacle immune to individual cognitive reframing. This structural environmental rigidity further clarifies why such demands bypass the anticipated buffering effect, compelling CSE must act independently to protect against burnout, rather than buffering the perception of the stressor itself.

Beyond the direct psychological strain, as this health impairment process persists, the negative impacts extend to trigger a chain reaction of cognitive and clinical impairment. Psychologically, severe cognitive depletion forces professionals to adopt defensive dehumanization mechanisms—manifested as Cynicism—fostering detachment in clinician-patient interactions (21, 44). Clinically, the attentional lapses and cognitive interruptions required to navigate cumbersome HIS/EMR interfaces significantly elevate the risk of data entry errors, missed medical orders, and compromised high-risk patient care (32, 33). Technostress represents not only an occupational health concern, but may also have implications for patient safety and the quality of care. During this transitional phase of digital governance, while structural reforms remain pending, fortifying personal resources like CSE provides a vital, immediate intervention entry point to safeguard healthcare delivery (7, 17, 21).

### 4.3 Implications for healthcare management and practice

Grounded in the JD-R framework, the independent dual-pathway mechanism calls for a recalibration of healthcare administration paradigms in healthcare administration. Rather than focusing solely on technical skill reinforcement, intervention priorities must shift toward fostering functional adaptation within high-pressure digital environments. This requires a coordinated approach spanning individual cognitive support, organizational resource allocation, and structural system optimization—addressing both the demands that deplete and the resources that energize the workforce.

At the individual level, strengthening personal resources is essential. Given that CSE functions as a key personal resource that directly inhibits burnout without altering the perception of objective technostress, healthcare administrators should invest in targeted psychological training tailored to clinical realities (45). The goal extends beyond system mastery: professionals must be guided to reframe technical hurdles—from administrative impositions into tools that ultimately support patient care. Such cognitive reframing may help activate the intrinsic motivational function of CSE. Beyond this, Mindfulness-Based Stress Reduction (MBSR) programs offer a complementary approach to counteract cognitive depletion. A recent meta-analysis of 16 randomized controlled trials in healthcare populations confirmed that mindfulness-based interventions significantly reduce burnout and strengthen psychological resilience (46).

Organizationally, the depletion of psychological resources caused by chronic hindrance demands necessitates systemic resource replenishment (18). One promising strategy is the implementation of Digital Mentorship programs, such as designating “Clinical Informatics Champions”—frontline professionals who take on the work of helping their colleagues navigate new systems. A scoping review found that such roles are most effective when champions are embedded in the clinical setting and backed by organizational support (47). This approach holds particular relevance given the intensive EMR use among frontline clinical staff. Drawing on vicarious experience (15), digitally proficient personnel can guide peers, reducing collective technical anxiety and enhancing group digital efficacy through observation and collaboration (48). Equally important is the cultivation of a psychological safety climate. Establishing non-punitive reporting mechanisms during system updates encourages all healthcare professionals to voice technical obstacles rather than conceal errors—alleviating psychological burden and buffering the cynicism associated with job burnout (49). Finally, given the observed negative correlation between income and technostress, performance-based financial incentives can serve as a pragmatic compensatory resource.

At the system level, adapting to poorly designed architecture should not become a perpetual cost borne by frontline clinicians. Reducing technostress at its source—by addressing structurally embedded job demands—may represent a more sustainable approach. Health administrations should promote Participatory Design by establishing clinically oriented feedback mechanisms that involve frontline professionals in the procurement, design, and iterative updating of medical information systems (39, 50). Resolving these objective hard constraints to ensure system architectures accommodate the unique, continuous nature of clinical documentation—a burden well-documented across healthcare professions (51)—while also serving the needs of physicians and other clinicians, offers the most sustainable path to reducing hindrance job demands. Only when technology aligns with the realities of clinical work can genuine human-technology synergy be achieved.

### 4.4 Limitations

This study is subject to several limitations. First, the cross-sectional design restricts the determination of causal relationships. Although the theoretical model draws upon the JD-R and COR frameworks, the observed findings regarding CSE, technostress, and job burnout remain correlational. Longitudinal designs or intervention studies are recommended for future research to clarify directional causality and temporal dynamics. Second, the exclusive use of quantitative methodology may overlook specific contextual nuances of digital adaptation in healthcare settings. Integrating qualitative approaches—such as interviews or observational studies—would better elucidate the organizational and individual mechanisms underlying technostress, offering a complementary perspective for future inquiry. Third, data collection relied on self-report measures, inevitably introducing risks of common method variance and social desirability bias. While statistical controls indicated no severe distortion in the present dataset, subsequent investigations would benefit from incorporating multi-source data or objective performance metrics to enhance validity. Finally, convenience and snowball sampling limited the ability to collect data from non-respondents, precluding a formal assessment of non-response bias. However, the inclusion of participants from multiple community healthcare settings with diverse demographic characteristics may partially alleviate concerns regarding representativeness. Future studies should employ probabilistic sampling and further extend the model by incorporating moderating or additional mediating variables to better capture the complexity of technostress processes in healthcare settings.

## 5 Conclusion

This study elucidates how CSE and technostress shape burnout among community healthcare professionals via independent routes. CSE serves as a personal resource that reduces exhaustion and cynicism, while technostress acts as a structural hindrance demand that directly aggravates burnout. Crucially, CSE does not buffer technostress—individual digital competence alone cannot overcome systemic constraints. Mitigating burnout thus requires a dual-track strategy: strengthening personal resources through training while prioritizing structural reforms that optimize system usability and reduce technological burden on frontline workers. Protecting healthcare professionals' occupational health is not only an workforce priority but a prerequisite for patient safety in an increasingly digitalized healthcare landscape.

## Data Availability

The raw data supporting the conclusions of this article will be made available by the authors, without undue reservation.
